# Instabilité post-traumatique du nerf ulnaire au coude: à propos de cinq cas et revue de la littérature

**DOI:** 10.11604/pamj.2016.24.297.9248

**Published:** 2016-08-03

**Authors:** Azzelarab Bennis, Adil Lamkhanter, Jalal Youssef, Mohammed Reda Ouzaa, Mohammed Benchakroun, Abdelouahab Jaafar

**Affiliations:** 1 Service de Traumatologie-Orthopédie I, Hôpital Militaire d’Instruction Mohammed V, Université Mohammed V, Rabat, Maroc

**Keywords:** Nerf ulnaire, instabilité, traumatique, Ulnar nerve, instability, traumatic

## Abstract

Le nerf ulnaire au coude a une situation anatomique très particulière, qui explique son importante vulnérabilité. Les auteurs rapportent cinq cas, d'instabilité post-traumatique du nerf ulnaire avec luxation complète du nerf en avant de l'épitrochlée. A la lumière de notre expérience et d'une revue de la littérature, les aspects pathogéniques, diagnostiques et thérapeutiques de cette pathologie sont passés en revue.

## Abstract (en)

At the elbow, the ulnar nerve has a peculiar anatomic location which explains its high vulnerability. We here report five cases of post-traumatic ulnar nerve instability with complete nerve dislocation in front of the epitrochlea. In the light of our experience and review of the literature, the pathogenic, diagnostic and therapeutic aspects of this pathology are reviewed.

## Introduction

La première description d'instabilité du nerf ulnaire au coude est rapportée par Childress en 1956, il s'agit d'une affection assez fréquente, le plus souvent d'origine congénitale [1]. Les formes post-traumatiques sont plus rares [2, 3]. Nous discutons, à partir de notre expérience et des données de la littérature, la pathogénie et les modalités diagnostiques et thérapeutiques de ces lésions.

## Méthodes

**Figure 1:**
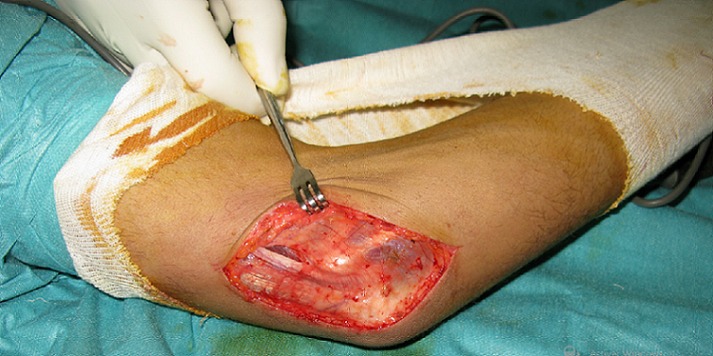
Vue peropératoire: nerf ulnaire dans sa gouttière à l’extension du coude

**Figure 2:**
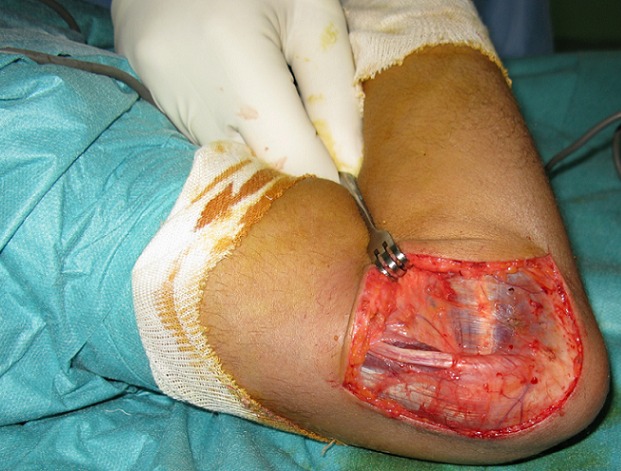
Vue peropératoire: luxation du nerf ulnaire en avant de l’épitrochlée à la flexion du coude

**Figure 3:**
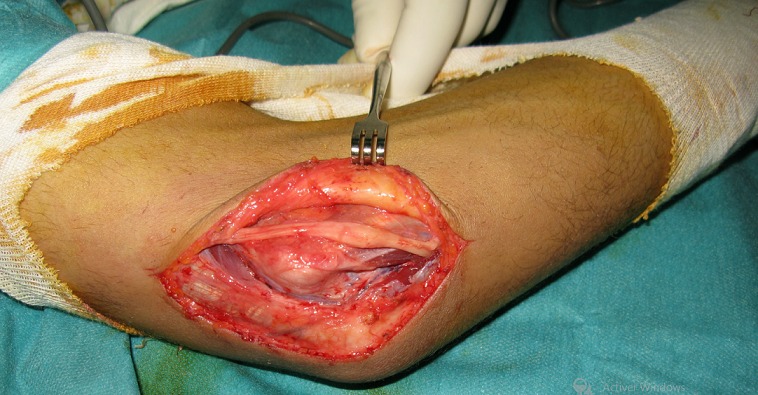
Vue peropératoire: aspect pseudo-nevromateux localisé du nerf ulnaire

Les auteurs rapportent une étude rétrospective de cinq cas d'instabilité post-traumatique du nerf ulnaire de type B, selon la classification de Childerss [1]. Il s'agit de 5 hommes, tous militaires de carrière. L'âge moyen était de 32 ans. Le côté droit était affecté 4 fois sur 5. La notion de traumatisme du coude avec impact direct sur son versant interne a été retrouvée chez tous les patients. Le diagnostic est posé un à deux ans après le traumatisme, lors d'une consultation spécialisée. La symptomatologie clinique était faite de douleurs gênantes du coude, à type de décharge électrique irradiant vers l'avant-bras et les doigts cubitaux, avec sensation d'un ressaut douloureux au niveau du coude à chaque mouvement de flexion-extension. La radiographie du coude était normale dans tous les cas. L'électromyogramme (EMG) révélait un ralentissement de la vitesse de conduction dans quatre cas. Tous nos patients ont bénéficié d'un traitement chirurgical, quatre ont été opérés d'emblée et un patient opéré 6 mois plutard après échec du traitement orthopédique. La transposition antérieure sous cutanée du nerf ulnaire a été réalisée chez 4 patients, alors que le cinquième patient a bénéficié d'un repositionnement du nerf dans sa gouttière après son évidement, associé à une plastie du rétinaculum du tunnel ulnaire à partir des aponévroses du triceps et des épitrochléens. L'exploration chirurgicale a révélé: une luxation récidivante du nerf ulnaire à la flexion-extension du coude dans tous les cas (Figure 1, Figure 2); une gouttière épitrochléo-olécranienne (E-O) peu profonde dans 4 cas et normale dans un cas; un rétinaculum très fin dans 2 cas, absent dans 2 cas et déchiré dans un cas; et un aspect pseudo-névromateux localisé du nerf dans 4 cas (Figure 3).

## Résultats

Le résultat post-opératoire était satisfaisant chez quatre patients, avec disparition complète du ressaut et des douleurs, et retour à la vie normale un mois après l'intervention. Le patient chez qui nous avons réalisé le repositionnent du nerf ulnaire dans sa gouttière, a présenté un syndrome canalaire, en rapport avec l'épaississement du rétinaculum réfectionné. Ce patient a été repris trois mois plus tard pour une transposition antérieure sous cutanée du nerf ulnaire, dont les suites étaient simples.

## Discussion

L'instabilité du nerf ulnaire au niveau du coude est une lésion assez fréquente, puisque son incidence a été estimée par Childress à 16,2% de la population normale [1]. Ce terme désigne une mobilité anormale du nerf ulnaire qui sort de la gouttière E-O et se mobilise. Il existe 2 types d'instabilité [1–4]: type A: le nerf se positionne à cheval sur le sommet de l'épitrochlée; type B: le nerf se luxe franchement en avant. Les instabilités post traumatiques du nerf ulnaire avec luxation complète du nerf en avant de l'épitrochlée sont rares [3]. La pathogénie de cette instabilité reste mal définie, car plurifactorielle [4]. En effet, lors de la flexion du coude, le triceps exerce une pression sur le nerf ulnaire qui le refoule en avant. Cependant, la translation antérieure du nerf ulnaire se trouve limiter par le relief osseux de l'épitrochlée et de son rétinaculum. La luxation du nerf ulnaire peut alors se produire par [5, 6]: un triceps proéminent ou l'instabilité de son chef interne; l'absence ou la laxité du rétinaculum qui ferme la gouttière E-O; la faible profondeur de cette gouttière; et le cubitus valgus. Les formes post-traumatiques peuvent être dues à une déchirure du rétinaculum ou à un remaniement du massif épitrochléen [7–9].

Dans notre série la dysplasie de la gouttière E-O et la déchirure de son rétinaculum étaient les principaux générateurs de cette instabilité. Cliniquement, l'instabilité du nerf ulnaire se manifeste par des douleurs chroniques du coude, à type de paresthésies irradiant le long du bord cubital de l'avant-bras et de la main, exacerbées à la flexion du coude avec sensation d'un ressaut, qui traduit la luxation du nerf. Une amyotrophie de la loge hypothénarienne peut également se voir en cas de névrite [5, 10–12]. Le syndrome douloureux et le ressaut du nerf étaient les signes d'appels chez nos patients. Les radiographies du coude peuvent être normales, ou révéler des remaniements traumatiques de l'épitrochlée [11]. L'échographie dynamique semble être plus intéressante dans les cas douteux, en montrant le ressaut du nerf ulnaire, associé parfois à celui du tendon du triceps selon Brasseur et Hammani [9]. L'imagerie par résonance magnétique (IRM) ne permet pas le diagnostic, mais évoque parfois la souffrance nerveuse, sous forme d'épaississement du nerf avec accentuation de l'hyposignal à sa périphérie [13]. L'EMG renseigne sur la névrite de friction, en montrant un ralentissement de la vitesse de conduction nerveuse [13, 14].

**Figure 4:**
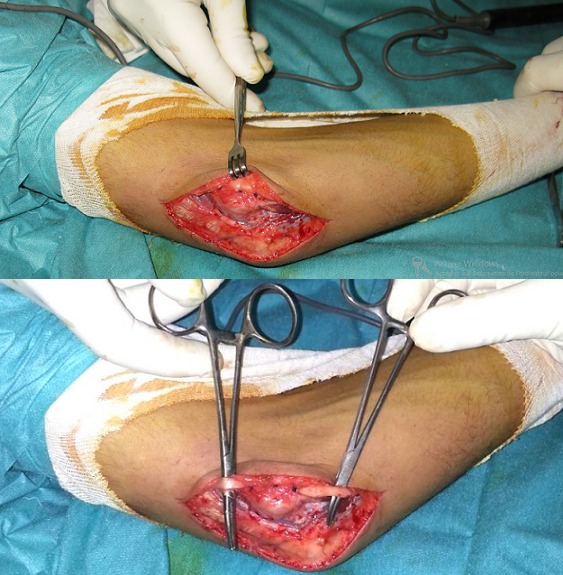
Vue peropératoire: transposition antérieure sous cutanée du nerf ulnaire

Le traitement de l'instabilité du nerf ulnaire de type B est chirurgical, en raison de la simplicité de l'acte opératoire et le retour rapide aux activités précédentes. Cette chirurgie est basée sur la transposition antérieure du nerf ulnaire, qui peut être sous cutanée, intramusculaire ou sous musculaire [1, 15]. D'autres techniques opératoires ont été également décrites, comme le repositionnement du nerf dans sa gouttière associé ou non à l'épicondylectomie médiale partielle [4–7]. Le traitement non chirurgical est indiqué chez les patients à symptomatologie positionnelle intermittente, il fait appel aux anti-inflammatoires et le port d'orthèse limitant la flexion du coude [16, 17]. Cependant, ce traitement reste astreignant et expose à la raideur du coude. Nous avons réalisé avec succès la transposition antérieure sous cutanée du nerf ulnaire (Figure 4) chez tous nos patients (quatre d'emblé et un patient après échec du repositionnement du nerf dans sa gouttières). Il s'agit d'une technique qui nous a donné entière satisfaction, elle représente aussi la tendance actuelle des chirurgiens [18].

## Conclusion

L'instabilité post-traumatique du nerf ulnaire avec luxation complète du nerf est une pathologie rare et handicapante par la névrite qu'elle entraine. Son diagnostic doit être précoce, il repose essentiellement sur la clinique. La transposition antérieure sous cutanée reste une technique simple et efficace.

### Etat des connaissances actuelle sur le sujet

- L'instabilité post-traumatique du nerf ulnaire au coude est une pathologie rare;
- Le diagnostic peut passer inaperçu en raison du contexte bénin du traumatisme;
- Pathologie douloureuse et handicapante.

### Contribution de notre étude à la connaissance

- Notre étude, rappelle une revue de la littérature de cette pathologie;
- Met l'accent sur la fiabilité de la technique de transposition antérieure du nerf ulnaire dans cette pathologie;
- Insiste sur l'intérêt du diagnostic et de traitement précoces, pour une évolution favorable.
